# 

**Published:** 1860-11

**Authors:** 


### LIST OF COLLABORATORS.

ARMOR, S. G., M.D.,
Late Professor of Pathology and Clinical Medicine, Missouri Medical College.

ATKINSON, WM. B., M.D.,
Lecturer on Obstetrics, and Diseases of Women and Children, Philadelphia.

ATLEE, JOHN L., M.D.,
Late President Pennsylvania State Medical Society.

ATLEE, WALTER F., M.D.,
Philadelphia.

BARCLAY, ROBERT G., M.D.,
Jerusalem, Syria.

BELL, JOHN, M D.,
Philadelphia, formerly Professor of Medicine, Medical College of Ohio.

BEMISS, S. M., M.D.,
Professor of Clinical Medicine, University of Louisville.

BLACKBURN, L. P., M.D.,
Natchez, Miss.

BLACKIE, GEORGE C., M.D.,
Professor of Botany, University of Nashville.

BLACKMAN, G. C., M.D.,
Professor of Surgery, Medical College of Ohio.

BOBBS, J. L., M.D.,
Formerly Professor of Anatomy, Indianapolis.

BOWEN, W. S., M.D.,
New York.

BOZEMAN, N., M.D.,
Montgomery, Ala.

BRADFORD, J. T., M.D.,
Augusta, Ky.

BRINTON, JOHN H., M.D.,
Lecturer on Operative Surgery, Philadelphia.

BUMSTEAD, FREEMAN J., M.D.,
New York.

CARNOCHAN, J. M., M.D.,
Professor of Surgery in the New York Medical College.

CHIPLEY, W. S., M.D.,
Professor of Medicine, Transylvania University.

CLAPP, A., M.D.,
New Albany, Indiana.

CLIFFE, D. B., M.D.,
Franklin, Tenn.

DA COSTA, J., M.D.,
Lecturer on the Practice of Medicine at the Philadelphia Medical Association.

DICKSON, SAMUEL H., M.D.,
Prof. of Medicine, Jefferson Medical College.

DUNGLISON, RICHARD J., M.D.,
Physician to the Burd Orphan Asylum, and to the Albion Society, Philadelphia.

DUPIERRIS, M., M.D.,
Havana, Cuba.

EDGAR, W. F., M.D.,
United States Army.

FARRAR, S. C., M.D.,
Jackson, Miss.

FENNER, C. S., M.D.,
Memphis, Tenn.

FISCHER, EMIL, M.D.,
Philadelphia.

FLINT, AUSTIN, M.D.,
Professor of Clinical Medicine, Auscultation, and Percussion, New Orleans School of Medicine.

GADBURY, W. Y., M.D.,
Benton, Miss.

GIBBS, O. C., M.D.,

Chautauque County, New York.

HALL, J. P., M.D.,
Glasgow, Ky.

HAMMOND, WM. A., M.D., U.S.A.,
Prof. of Anat. in the University of Maryland.

HARDIN, JOHN, M.D.,
Professor of Obstetrics, Kentucky School of Medicine, Louisville.

HARTSHORNE, EDWARD, M.D.,
Surgeon to the Pennsylvania Hospital.

HASKINS, E. B., M.D.,
Professor of Medicine, Shelby Medical College, Nashville, Tenn.

HENTZ, C. A., M.D.,
Marianna, Florida.

HEWSON, ADDINELL, M.D.,
One of the Surgeons to Wills Hospital for Diseases of the Eye, Philadelphia.

HOLLINGSWORTH, SAMUEL L., M.D.,
Formerly Editor of the Medical Examiner, Philadelphia.

JEWELL, WILSON, M.D.,
Philadelphia, formerly President of the Quarantine and Sanitary Convention.

KEATING, WM. V., M.D.,
Lecturer on Obstetrics and the Diseases of Women, at the Phila. Medical Association.

KERR, JOHN G., M.D.,
Canton, China.

KUNKLER, G. A., M.D.,
Madison, Ia.

LOGAN, BEN., M.D.,
Shreveport, La.

LOPEZ, A., M.D.,
Mobile, Ala., formerly President of the Alabama State Medical Society.

MEIGS, J. AITKEN, M.D.,
Professor of the Institutes of Medicine in the Medical Department of Penna. College.

MITCHELL, S. WEIR, M.D.,
Lecturer on Physiology at the Philadelphia Medical Association.

MOREHOUSE, GEORGE R., M.D.,
Philadelphia.

PACKARD, JOHN H., M.D.,
Philadelphia.

RAPHAEL, B. I., M.D.,
Professor of the Principles and Practice of Surgery and Surgical Pathology, in the New York Medical College and Charity Hospital.

REEVE, J. C., M.D.,
Dayton, Ohio,

REEVES, JAMES E., M.D.,
Fairmount, Virginia.

RIVES, L. C., M.D.,
Formerly Professor of Midwifery, Medical College of Ohio.

SMITH, J. LAWRENCE, M.D.,
Professor of Chemistry, University of Louisville.

SNEED, W. C., M.D.,
Frankfort, Ky., formerly President of Kentucky State Medical Society.

SPILMAN, C. H., M.D.,
Harrodsburg, Ky., formerly President of Kentucky State Medical Society.

STORER, HORATIO R., M.D.,
Formerly one of the Physicians to the Boston Lying-in Hospital, Boston, Mass.

SUTTON, GEO., M.D.,
Aurora, Ia.

SUTTON, W. L., M.D.,
Georgetown, Ky., formerly President Kentucky State Medical Society.

THOMPSON, S., M.D.,
Albion, Ill., formerly President of the Illinois State Medical Society.

TODD, L. BEECHER, M.D.,
Lexington, Kentucky.

WILLIAMS, E., M.D.,
Cincinnati, Ohio.

### Bi-Monthly Bibliographical Record.

### AMERICAN.

An Elementary Treatise on Human Anatomy. By Joseph Leidy, M.D., etc. 8vo., pp. 663. Philadelphia: J. B. Lippincott & Co., 1860. (From the publishers.)

Memoranda Medica; or, Note-Book of Medical Principles. By Henry Hartshorne, M.D. 12mo., pp. 190. Philadelphia: J. B. Lippincott & Co., 1860. (From the publishers.)

A Treatise on Fever. By Rezin Thompson, M.D. Third edition. 12mo., pp. 448. Nashville, 1860. (From the author.)

A Case of Removal of the Lower Jaw for Osteo-Sarcoma of immense size. By G. C. Blackman, M.D. Reprint, pp 8. Cincinnati, 1860. (From the author.)

Physician's Hand-Book of Practice, for 1861. By William Elmer, M.D. pp. 200. New York: W. A. Townsend & Co., 1860. (From the publishers.)

The Physician's Visiting List, Diary and Book of Engagements, for 1861. Philadelphia: Lindsay & Blakiston, 1860. (From the publishers.)

### FOREIGN.

Du Traitement par L'Uréthrotomie interne des Rétrécissements Organiques de l'Uréthre, Réfractaires aux Moyens Ordinaires. Thèse pour le Doctorat en Médecine. Par Felix-Eugene Dupierris, M.D. 4to., pp 78. Paris: Rignoux, 1860. (From the author.)

Further Researches on the Gray Substance of the Spinal Cord. By J. Lockhart Clarke, F.R.S. 4to., pp. 31. London: Taylor & Francis, 1859. (From the author.)

The Wife's Domain. By Philothalos. 12mo., pp. 162. London: Churchill, 1860. (From the author.)

The Science of Life, Health, and Disease. By Charles Searle, M.D., etc. 8vo., pp. 284. London: Booth, 1860. (From the author.)

Skin Diseases, and their Remedies. By Robert J. Jordan, M.D., etc. 12mo., pp. 283. London: Churchill, 1860. (From the author.)

The Diseases, Injuries, and Malformations of the Rectum and Anus; with Remarks on Habitual Constipation. By T. J. Ashton, M.D., etc. Third edition. 8vo., pp. 420. London: Churchill, 1860. (From the author.)

Theory and Practice of Midwifery. By Fleetwood Churchill, M.D., etc. 8vo., pp. 655. New American edition. Philadelphia: Blanchard & Lea, 1860. (From the publishers.)

The Principles and Practice of Modern Surgery. By Robert Druitt, M.D., etc. New American edition. 8vo., pp. 695. Philadelphia: Blanchard & Lea, 1860. (From the publishers.)

Des Lésions des Ganglions Lymphatiques Visceraux. Par C. Potain, M.D., etc. 8vo., pp. 81. Paris: Adrien Delahaye, 1860.

Commentary on the Hindu System of Medicine. By T. A. Wise, M.D., etc. 8vo., pp. 431. London: Trübner & Co., 1860.

Des Maladies de Croissance. Par Raoul Regnier, M.D. 8vo., pp. 102. Paris: Adrien Delahaye, 1860.

### INDEX.

Abdomen, colloid tumors of, 866.
general phlegmon of, 757.
Abscess, of heart, 755.
of kidney, 757.
of prostate gland, 460.
psoas, 115.
Acupressure, 308, 552, 570.
Adams, A. D., M.B., on rupture of the spleen, 756.
Adams, F., M.D., on fœtal auscultation, 364.
Addison, Dr., necrological notice of, 756.
Adler, J. M., M.D., on foreign body in bronchus, 1062.
Albumen, mode of estimating quantity of in fluids, 746.
Albuminous kidney, 129.
Alcohol, normal presence of in blood, 144.
Aldis, C. J. B., M.D., on paralysis of the right side, 711.
Aldridge, J. H., M.D., on rupture of uterus, 1087.
Aleurites tribola, oil of, 348.
Aloes, tincture of, in gleet, 345.
Althaus, J., M.D., treatise on medical electricity by, review of, 242.
Alum lozenges in affections of the throat, 345.
Alum, in the treatment of uterine hemorrhage, 717.
American Medical Association, 187.
proceedings of, 766.
transactions of, notice of, 639.
American Medical Biography, 956.
literature abroad, 957.
Ammonia, carbonate of, in chronic bronchitis, 539.
muriate of, in nervous cephalalgia, 541.
Ammonium, iodide of, in constitutional syphilis, 349.
Amputation, at hip-joint, 123, 880.
cases of, with acupressure, 552.
secondary, after gunshot wounds, 704.
spontaneous, below knee, 91.
tibio-tarsal, 505.
Anatomy of human lung, treatise on, by Waters, notice of, 837.
Anatomy, surgical, treatise on, by Malgaigne, notice of, 822.
Anæmia, rapidly fatal, 134.
Ancellon, Dr., on treatment of staphyloma by the ligature, 883.
Ancient Medicine, 1125.
Aneurism, aortic, 132.
femoral, cured by compression, 484.
iliac, ligation of common iliac for, 122.
iliac, fatal by rupture into vagina, 935.
inguino-femoral, cured by compression, 124.
traumatic, of anterior tibial, 79.
treatment of, by digital compression, 311, 1070.
Angina, membranous, garlic and lemon-juice in, 347.
Ansaloni, Dr., on the solanaceæ in tenesmus, 348.
Antipyretic remedies, efficacy of, 914.
Anus, fistule of, 270.
Aorta, abdominal, aneurism of, 132.
contraction and obliteration of, 713, 753.
imperforate arch of, and rupture of root of, 932.
Aortic valves, rupture of, 131.
slit-like perforation of, 932.
Aphonia, syphilitic, 882.
Apoplectic affections of retina, 1081.
Appendix cæci, discharged from bowels, 135.
Argenti, Dr., on alum in throat affections, 345.
Army Medical School, 762.
Army and navy dentists, 187.
Arsenic eaters of Styria, 920.
fallacy of sources of, in dead bodies, 350.
in apoplectic congestion, 1099.
in chorea, 160.
in menorrhagia, 138.
poisoning by, 747, 1111.
Arsenical fly-papers, 550.

Arsenical poisoning by paper, 550.
Arseniuretted hydrogen, inhalation of, 165.
Arteries, transposition of, 113.
Artery, coronary, rupture of, 303.
femoral, ligation of, 703.
iliac, common, ligation of, 122, 874.
iliac, internal, aneurism of, 935.
Asarum Europæum, for drinking, 1099.
Ascarides, treatment of, 540.
Ashton, T. J., M D., treatise on rectum and anus by, notice of, 1021.
Asphyxia neonatorum, mouth to mouth method in, 332.
Asthma, injection of atropine on the pneumogastric nerve for, 343.
Asylums, private, for the insane, 376.
Atheromatous expression, 712.
Atkinson, W. B., M.D., on intra-uterine fracture of clavicle, 509.
Atlee, W. L., M.D., on diagnosis of ovarian tumors, 330.
Atropine, sulphate of, injection of, in asthma, 343.
sulphate of, injection of, in tetanus, 738.
Auricle, right, of heart, rupture of, 132.
Auscultation, fœtal, 364, 525.
Ayre, J., M.D., necrological notice of, 383.
Ayres, H. P., M.D., on dysentery, 842.

Bagot, Dr., on the sickness of pregnancy, 136.
Baker, W., M.D., on double transverse hemiplegia, 1080.
Barallier, Dr., on muriate of ammonia in nervous cephalalgia, 541.
Barker, F., M.D., necrological notice of, 191.
Barker, T. A., M D., on imperforate arch and rupture of the root of the aorta, 932.
Barnes, R., M.D., on retroversio uteri in labor, 325.
Barrett, J. P. M.D., necrological notice of, 191.
Barthez, Dr., on arsenious acid in chorea, 160.
Barwell, R., M.D., on osteitis and inflammation of articular cartilage, 936.
Batley, R. M.D., on vesico-vaginal fistule, 313.
Bauer, L., M.D., on recto-vesical lithotomy, 121.
translation of Van Düben on microscopical diagnosis by, notice of, 263.
Baumgarten, G. H. E., M.D., on Virchow's views on cellular pathology, 518.
Beale, L., M.D., on how to work with the microscope, notice of, 1024.
Beck, T. R., M.D., and J. B., M.D., elements of medical jurisprudence by, review of, 433.
Becquerel, Dr., on tannin in uterine affections, 539.
Bedford, G. S., M.D., clinical lectures on the diseases of women and children by, notice of, 448.
Belladonna, opium as an antidote to, 285.
Bell and Watson, Drs., on extraction of cataract by linear incision, 174.
Bellinger, Dr., necrological notice of, 1134.
Bennett, J. H., M.D., clinical lectures on the principles and practice of medicine by, notice of, 449.
Berard, Dr., on the inhalation of chloroform, 544.
Bergouhnioux, Dr., on the green or blue color of pus, 162.
Bernard, C., M.D., on the cause of death in animals subjected to high temperatures, 338.
Berthelot and De Luca, Drs., on sugar found in the liver, 163.
Bevan, P., M.D., on scalds of the larynx, 501.
Bigelow, J., M.D., treatise on nature in disease by, notice of, 643.
Births and deaths in Philadelphia, 956.
Bismuth, subnitrate of, in burns and scalds, 655.
tannate of, in diarrhœa, 347.
Bladder, absence of, 758.
cancer of, 493.
stone in, 84, 312, 352, 466, 875.
Blenorrhagia, opiated wine of colchicum in, 346.
Blind, dictionary for, 1131.
Blondlot, Dr., on poisoning by arsenic, 747.
Blood, treatise on circulation of, in the head and extremities of man, by Sucquet, notice of, 840.
coagulation of, 149, 334.
in venous system during life, 511.
normal presence of alcohol in, 144.
red corpuscles of, action of salts on while circulating, 143.
Blood-crystals, their judicial medical importance, 163.
Blood-disease, treatise on by Hughes, review of, 427.
Blood-stains, detection of, 551, 1108.

Bloxam, Prof., on electrolysis in detection of metallic poisons, 747.
Boedeker, C., M.D., on estimation of quantity of albumen in albuminous fluids, 746.
Bone, reproduction of, 904.
Borax in diphtheria, 345.
Bourgeois, D., on rupture of the uterus during pregnancy, 721.
Bourgignon, Dr., on glycerin ointment for the itch, 160.
Bouvier, Dr., on woorara, 343.
Bowman, W., on iridectomy in glaucoma, 1069.
Boyer, P. M.D., necrological notice of, 191.
Boylston medical prizes, 1132.
Bozeman, N., M.D., on button suture for varicose veins, 877.
Bradford, J. T., M.D., on ovariotomy, 502.
Braid, J., M.D., necrological notice of, 767.
Brain, capillaries of, 149.
  concussion of, 649.
    with laceration, 647.
  diseases of, in children, iodide of potassium in, 527.
  obscure diseases of, treatise on by Winslow, review of, 1003.
  softening of, 306.
  tumor in fourth ventricle of, 318.
Braithwaite's Retrospect, 382.
  an epitome of by Wells, notice of, 452, 1025.
Braun, C., M.D., on the production of premature labor, 325.
Breast, cancer of, rapidly recurring, 86.
  scirrhus of, 281.
  tumor of, 119.
Brent, G. S., M.D., on enlargement of thymus gland, 1091.
Brett, R. H., M.D., on arsenical fly-papers, 550.
Bright's disease of the kidneys, treatise on by Frerichs, review of, 961.
Bright's kidney, 116, 130.
Brinton, Dr., on extra-capsular fracture of femur, 496.
  on fracture of spine, 492.
Briquet, Dr., on chloroform in hysteric paroxysm, 332.
Broca, P., M.D., on congenital inequality of the two sides of body, 509.
Brodie, Sir Benjamin, case of, 1130.
Bromine in pseudo-membranous croup, 161.
  Ozanam's solution of, formula for, 744.
Bronchial muscles, paralysis of, 884.
Bronchitis, chronic, carbonate of ammonia in, 539
Bronchocele of enormous size, 88.
Bronchus, foreign body in, 1062.
Brown, J. B., M.D., on retained menses from imperforate os uteri, 897.
  on vesico-vaginal fistula, 313.
Brown, Dr., on suction method of rearing infants, 142.
Browne, Dr., on chlorodyne, 915.
Brown-Séquard, E., M.D., appointment of, 765.
  on exciting influence of light, cold and heat on the iris, 148.
  on paralysis of the lower extremities, 708.
  on production of epilepsy in guinea-pigs, 337.
Brücke, Dr., on detection of blood-stains, 551.
Bruns, Dr., on treatment of cystic goitre, 707.
Bryan, J., M.D., an essay on hernia by, notice of, 265.
Bryant, T., M.D., on hemorrhage as a sign of polyp of rectum, 333.
  treatise on disease of joints by, review of, 810.
Buchanan, A., M.D., on lithotomy as a cause of death, 700.
Buchanan, A. H., M.D., on amputation at the hip-joint, 123.
Buchanan, M. S., M.D., necrological notice of, 959.
Buchner and Simon, Drs., on blood crystals, 163.
Budd, G., M.D., on hydatid tumor of heart, 754.
Burnett, C. M., M.D., on diagnosis and prognosis of mental diseases, 1077.
Burns, A. P., M.D., on arsenic in menorrhagia and leucorrhœa, 138.
  cherry-laurel water in, 539.
  and scalds, subnitrate of bismuth in, 655.
Busch, Prof., observations on digestion by, 724.
Butler, S. W., appointment of, 186.
Buzzell, J. M., M.D., on a fœtus carried twenty-two months beyond term, 894.
Byrne, Dr., necrological notice of, 1134.

CABOT, DR., on tumor of kidney, 758.
Caffein as an antidote to opium, 918, 1023.
Calculus, renal, 1067.
  vesical, 1074.
Caloric, treatise on by Metcalfe, notice of, 1019.
Campbell, H. F., M.D., on caffein as an antidote to opium, 918, 1023.
  on quinine in croup, 903.
Cancer, epithelial, actual cautery in, 126.
  of bladder, 493.

Cancer of breast, 86.
of heart, 493, 931.
of liver, lungs, and spleen, 297.
of mesentery and duodenum, 293.
of stomach, 1083.
Cancerous degeneration of lung, 1115.
Cap, Dr., on tannate of bismuth in diarrhœa, 347.
Capillaries of the brain, 149.
Carnochan, J. M., M.D., on tic douloureux, 506.
Cartilage, articular, pathology of, 936.
Casper, Dr., on nitro-benzine, 548.
Cataract, congenital, 73.
extraction of, by linear incision, 174.
Cautery, actual, in epithelioma, 126.
in incontinence of urine, 453.
Cazin, Dr., on garlic and lemon-juice in membranous angina, 347.
Cephalalgia, nervous, muriate of ammonia in, 541.
Cephal c version of fœtus in utero by external manipulation, 559.
Cerebellum, pathology of, 513.
Cerebral symptoms independent of cerebral disease, 320.
substance, composition of, 747.
Charity Hospital, selected cases from, 646.
Chelidonium majus in prurigo, 348.
Cherry-laurel in burns, 539.
Chest, diseases of, treatise on by Gerhard, notice of, 260.
Chevallier, A., M.D., on Schweinfurt green, 1109.
Chew, S., M.D., on paralysis of the lower extremities, 711.
Chicago College of Pharmacy, 186.
Chicago Medical Examiner, 186.
Child, G. W., M.D , on chorea and acute rheumatism, 1076.
Childhood, mental peculiarities and disorders of, 526.
Children's Hospital of Philadelphia, 782.
Chinese medicines, 346.
Chlorine lotions in variola, 537.
Chlorodyne, properties of, 915.
Chloroform, death from, 351.
Faure's method of inhaling, 544.
in hooping-cough, 159.
in hysteric paroxysm, 332.
in puerperal convulsions, 895.
Chordæ tendineæ, rupture of, 131.
Chorea, arsenious acid in, 160.
Chorea and acute rheumatism, connection between, 1076.
Clapp, W. A., M.D., on compression treatment of aneurism, 484.
Clavicle, dislocation of both ends of, 314.
Clavicle, dislocation of, treated by wiring, 1071.
intra-uterine fracture of, 509.
Cleland, Dr., on saccharated lime, 547.
on diseases of uterus, 1116.
Clemens, T., M.D., on a substitute for Fowler's solution, 158.
Clinics of the Jefferson Medical College, 731, 1053.
Philadelphia Hospital, 266, 453.
Coagulation of the blood in the venous system during life, 511.
researches on, 334.
Coca, a new alkaloid in, 1105.
Coccygeal gland of man, 734.
Cockle, Dr., on aneurism of internal iliac, 935.
Cod-liver oil, history of, 1102.
Coffee, infusion of, in strangulated hernia, 541.
Colchicum, opiated wine of, in rheumatism, 916.
and opium in blenorrhagia, 346.
saccharated wine of, in gout and rheumatism, 1100.
Cole, R. B., M.D., on alum in uterine hemorrhage, 717.
Colitis, chronic, 95.
Colloid tumors of the abdomen, 866.
Collyrium in ophthalmia of new-born infants, 345.
Compression treatment of aneurism, 124, 484, 1070.
of gleet, 869.
Concussion of brain, 647, 649.
Congenital inequality of the two sides of the body, 509.
Consumption, tubercular, importance of functions of skin in, 713.
radical cure of, 955.
Cornelian cherry, notes on, 534.
Coronary artery, rupture of, 303.
Corvisart, L., M D., on a little-known function of the pancreas, 528.
on pepsin in the vomiting of pregnancy, 739.
Coryza, chronic, powder for, 347.
Coup de soleil, remarks on, 859.
Courty, Prof., on sulphate of atropine in asthma, 343.
Coxo-femoral disarticulation in relation to military surgery, 939.
Creosote in cough and purulent expectoration, 158.
Crichton, Dr., necrological notice of, 1134.
Crimea, notes on surgery of war in, by Macleod, review of, 30.
Croft, R. C., M.D., on diagnosis between pregnancy and abdominal tumors, 523.
Croup, quinine in, 903.

Croup, tracheotomy in, 165, 505, 854.
Curare in tetanus, 344.
Cutter, E., M.D., on attempts to produce variola by inoculating with virus of true variola, 716.
Cyst, serous, of neck, 81.
Cystic disease of testicle, 76.

DALTON, DR., on rupture of the chordæ tendineæ, 131.
Davy, Dr., on the coagulation of the blood, 149.
Deafness, ether for, 1101.
Death and camphine, 378.
  cause of in animals subjected to high temperatures, 338.
  sudden, 887.
Debout, Dr., on syncope from surgical hemorrhage, 703.
Debrou, Dr., on subungual exostosis, 876.
Delattre, Dr., on oleum morrhuæ, rajæ, and squali, 150.
Delirium, hypochondriacal, a form of, 716.
  tremens, rational treatment of, 945.
Démarquay, Dr., on glycerole of tannin in vaginitis, 898.
  on fibrous tumors of the uterus, 899.
  on glycerin, 155.
Dental colleges, 565.
Dentistry, operative, treatise on by Taft, review of, 414.
Denton, Dr., necrological notice of, 1134.
D'Etoilles, Dr., necrological notice of, 1134.
Denuncé, Dr., on digital compression in aneurism, 311.
Devergie, A., M.D., on homicidal mania, 133.
  on iodide of the chloride of mercury in skin diseases, 535.
Diathesis, pyogenic or suppurative, 99.
  scrofulous, 885.
Diarrhœa, tannate of bismuth in, 347.
Dictionary for the blind, 1131.
Diday, Dr., on syphilitic aphonia, 882.
Digitaline in puerperal fever, 160.
Digestion, observations on, 724.
Digital compression in aneurism, 311, 1070.
Diphtheria, borax in, 345.
  inoculability of, 379.
  paralysis consequent upon, 710.
Disinfectants, 1118.
Dislocation, lateral, of both knees, 310.
  of both ends of clavicle, 314.
  of clavicle treated by wiring, 1071.
  of head of femur into thyroid foramen, 646.
Displacements of the uterus, 722.
Dixon, Dr., on apoplectic affections of retina, 1081.
Dropsy, employment of lambucus nigra in, 344.
Drowning, researches on death by, 1109.
Druitt, R., M.D., on fœtal auscultation, 525.
  treatise on surgery by, notice of, 1022.
Ducachet, Dr , on morbid growths of the ventricles of the larynx, 113.
Duncan, J. F., M.D., on paralysis of the bronchial muscles, 884.
Dunglison, Prof., on rational treatment of delirium tremens, 945.
Dunglison, R. J., M.D., on statistics of insanity in the United States, 656.
  appointment of, 1133.
Dunn, Dr., on records of midwifery practice, 138.
Dunton, W. R., M.D., on abnormal position of kidney, 935.
Duodenitis, chronic, 95.
Dura mater, inflammation of sinuses of, 1073.
Dysentery, its pathology, causes, and treatment, 842.

EAR, diseases of, treatise on by Toynbee, review of, 769.
Eclampsia in a primipara, 862.
Eisenmann, Dr., on blenorrhagia, 346.
  on chlorine lotions in variola, 537.
  on opiated wine of colchicum in rheumatism, 916.
Electricity, medical, treatise on by Althaus, review of, 242.
Elmer and Elsberg, physician's handbook of practice by, notice of, 71.
Elwell, J. J., M.D., treatise on malpractice by, notice of, 832.
Embolie of cerebral artery, 1111.
Enteric fever, treatise on by Reeves, review of, 57.
Epilepsy, case of, 930.
  production of guinea-pigs, 337.
  selinum palustre in, 348.
  with ossification of falx cerebri, 316.
Epithelial cancer, actual cautery in, 126.
Erythroxylon coca, medical properties of, 341.
Ether, a remedy for deafness, 1101.
  sulphuric, as an anæsthetic, 172.
Excision of the hip-joint, 706.
  of elbow-joint, 1073.
  of knee-joint, 1072.
Exhaustion after suckling, 327.
Exodus of Southern medical students, 188.

Exostosis, subungual, 876.
Evans, C., M.D., on tracheotomy in croup, 165.

FEMORAL artery, aneurism of, 484.
ligation of, 79, 703.
Femur, dislocation of head of into thyroid foramen, 646.
Fenner, C. S., M.D., on tracheotomy in croup, 854.
Fever, enteric, treatise on by Reeves, review of, 57.
Fifield, W. C., M.D., on ovarian tumor simulating pregnancy, 331.
Fischer, Dr., on cutaneous eruption, following use of iodine, 743.
Fiske Fund prizes, 1132.
Fistule, gastric, cured by plastic operation, 708.
of anus, 270.
œsophageal, 517.
vesico-vaginal, new operations for, 313.
Fistulous ulcer of larynx, 879.
Flehinger, Dr., on chloroform in hooping-cough, 159.
Flint, A., M.D., treatise on diseases of heart by, review of, 1.
Flourens, Dr., on coloration of fœtal bones by madder, 1093.
Fœtal auscultation, 364, 525.
Fœtus in utero, influence of mother's mind on, 521.
carried twenty-two months beyond term, 894.
coloration of bones by madder, 1093.
Ford, W. H., M.D., on alcohol in the blood, 144.
Forget, Prof., on influence of warm climates on phthisis, 887.
Fornix and septum lucidum, softening of, 497.
Foucher, Dr., on a collyrium for ophthalmia of new-born infants, 345, 724.
Fountain, E. J., M.D., on syrup of phosphates in stomatitis materna, 89.
Fourtoul, Dr., on ophthalmia of new-born infants, 903.
Fowler's solution, a substitute for, 158.
Fox, W. T., M.D., on phlegmasia dolens, 1089.
Foy, Dr., on hæmatic capsules, 160.
Fracture, compound, of skull, 651.
extra-capsular, of neck of femur, 496.
intra-uterine, of clavicle, 509.
of anatomical neck of humerus, 118,
of neck of scapula, 882.
of the spine, 492.
spasmodic, of both femurs, 1072.
transverse, of the patella, 286.
Fractures and dislocations, treatise on by Hamilton, review of, 577.
Francis, S. W., M.D., report of Mott's surgical clinic by, notice of, 839.
Franchino, Dr., on cherry-laurel water in burns, 539.
French medical literature, curiosities of, 377.
mill-stone makers, phthisis of, 514.
Frerichs, Dr., treatise on Bright's kidney by, review of, 962.
Frick, C., M.D., necrological notice of, 573.
Frosmuller, Dr., on Indian hemp, 739.
Fuller, Dr., on cancer of heart, 931.
on hooping-cough, 1092.

GAFFARD, DR., on excessive perspiration of the feet, 540.
Gall-stones, pathology and treatment of, 177.
Gamberini, Dr., on iodide of ammonium in constitutional syphilis, 349.
on tincture of aloes in gleet, 345.
Ganglions of wrist, 82.
Gangrene of the uterus and placenta, 721.
senile, 472.
Garrod, A. B., M.D., treatise on gout by, review of, 988.
Gastric fistule, cured by plastic operations, 708.
Gastritis, chronic, 95.
Gelsemin, observations on, 743.
Gerhard, W. W., M.D., treatise on diseases of chest by, notice of, 260.
Gibb, Dr., on albuminous and saccharine kidneys, 129.
on the atheromatous expression, 712.
on sanguinaria canadensis, 542.
Gillette, Dr., necrological notice of, 191.
Gland, coccygeal, of man, 734.
prostate, abscess of, 460.
Glaucoma, acute, Hancock's operation for, 128.
iridectomy in, 1069.
Gleet, compression treatment of, 869.
tincture of aloes in, 345.
Globularia alypum, medical properties of, 534.
Glycerin, its medical and surgical uses, 155.
in preparation of sinapisms, 738.
ointment for itch, 160.
Goitre, cystic, treatment of, 707.
Gold-headed cane, 182, 369, 948.
Gold, muriate of, in affections of stomach and chronic vomiting, 159.
Goldsmith, M., M.D., on uterine polyps, 901.
Gore, W. R., M.D., on compression treatment of aneurism, 124.

Gosebruck, Dr., on arseniuretted hydrogen, 165.
Gosselin, Dr., on the taxis in strangulated hernia, 309.
Gout and rheumatic gout, treatise on by Garrod, review of, 988.
Grand-Clement, Dr., on chelidonium majus, in prurigo, 348.
Granulations of lining membrane of uterus, 328.
Gray, Dr., on caries of spine, 1079.
Grimault, Dr., on glycerin in preparation of sinapisms, 738.
Gross, Prof. S. D., M.D., clinics by, 73, 266, 453, 1053.
 on abscess of the prostate gland, 460.
 on anal fistule, 270.
 on aneurism of anterior tibial, 79.
 on cancer of breast, 86.
 on cancer of the heart, 493.
 on colloid tumors of abdomen, 866.
 on congenital cataract, 73.
 on cystic disease of testicle, 76.
 on ganglions of wrist, 82.
 on lateral operation of lithotomy, 84, 466.
 on neuralgia of the stump, 283.
 on origin of ovariotomy, 1028.
 on paralysis of inferior straight muscle of eye, 477.
 on polyp of rectum, 84.
 on prostatorrhœa, 693.
 on pyæmia, 469.
 on senile gangrene, 472.
 on serous cyst of neck, 81.
 on spina bifida, 1053.
 on spontaneous cure of scirrhous breast, 281.
 on stricture of the urethra, 467.
 on the actual cautery in incontinence of urine, 453.
 on tumor of labium, 266.
 on universal melanosis, 488.
Gross, S. W., M.D., appointment of, 955.
Guirette, Dr., on radical cure of consumption, 955.
Guislan, Dr., necrological notice of, 767.
Gullard, A. D., M.D., on hydatid tumor and abscess of liver, 756.
Guthrie, C. G., M.D., necrological notice of, 191.

HABERSHON, S. D., M.D., treatise on mercury by, review of, 632.
Hachenberg, G. P., M.D., on compression in gleet, 869.
Hæmatic capsules, 160.
Hake, T. G., M.D., on the scrofulous diathesis, 885.
Hamilton, F. H., M.D., treatise on fractures and dislocations by, review of, 577.
Hamilton, J. W., M.D., on ovariotomy, 127.
 on dislocation of both knees, 310.
Hancock, Dr., on the treatment of acute glaucoma, 128.
Hardaway, Dr., records of midwifery practice by, 138.
Hardee, W., M.D., on infantile convulsions, 1093.
Hardey, R., M.D., on position and binder in impeded parturition, 521.
Hargis, R. B. S., M.D., on eclampsia, 862.
Harlan, Dr., on cancer of the mesentery and duodenum, 293.
 on rupture of the coronary artery, 303.
 on typhoid fever, 305.
Harris, J., M.D., on fracture of neck of scapula, 882.
Harrison, Dr., records of midwifery practice by, 137.
Hayes, Dr., on abscess of the kidney, 757.
Hayward, G., M.D., on ether as an anæsthetic, 172.
Headland, F. M., M.D., treatise on action of medicines by, notice of, 71.
Heart, abscess of, 755.
 cancer of, 493, 931.
 disease of, treatise on by Flint, review of, 1.
 right auricle of, rupture of, 132.
 right ventricle of, hydatid tumor of, 754.
 wound of, 496.
Hecker, Prof., on hydrostatic test, in still-born children, 349.
Heisch, C., M.D., on the arsenic eaters of Styria, 920.
Hellebore, the different species of, 151.
Heller, Dr., on iodized oil of juniper-berries, 739.
Hemiplegia, double transverse, 1080.
Hemorrhage, cerebellar, effect of on muscular motions, 319.
 from bowel, a symptom of polyp of rectum, 333.
 post partum, its prevention, 324.
  its treatment, 520.
 uterine, alum in, 717.
Hemorrhagic diathesis, 1082.
Hemorrhoids and prolapse of rectum, treatise on by Smith, notice of, 644.
Henderson, W., M.D., on movable kidney, 129.
Henfrey, Dr., necrological notice of, 191.
Hernia, an essay on by Bryan, notice of, 265.

Hernia, strangulated, memoir on by Macilwain, notice of, 450.
infusion of coffee in, 541.
taxis in, 309.
Herpin, Dr., on selinun palustre in epilepsy, 348.
Hervieux, Dr., on treatment of ascarides, 540.
Hewitt, G., M.D., on vesicular mole, 141.
Hicks, J. D., M.D., on version in abnormal labor, 1085.
Hillairet, Dr., on effect of cerebellar hemorrhage on muscular motion, 319.
Hip-joint, amputation at, 123, 880, 939.
excision of, 706.
Hirsch, Dr., on creosote in coughs and purulent expectoration, 158.
Hobson, H., M.D., surgery of the Western Nations by, notice of, 72.
Hodge, L., M.D., on fracture of the humerus, 118.
on fused kidneys, 488.
on hypertrophy of prostate gland, 492.
on incised wound of intestines, 306.
on tumor of breast, 119.
Hodgen, J. T., M.D., on wiring dislocated clavicle, 1071.
Hoffman, F., M.D., on detection of phosphorus, 549.
Hogg, J., M.D., on dislocation of crystalline lens, 1074.
Homicidal mania, 133.
Honors to medical men, 1130.
Hooping-cough, chloroform in, 159.
sulphate of zinc in, 1092.
Hoppe, Dr., on muriate of gold in vomiting and gastritis, 159.
on tinctura of thuiæ in bad smells of nose, 915.
Howard Hospital, 955.
Howard, R. P., M.D., on myeloid tumors, 877.
Hughes, J. V., M.D., treatise on blood-disease by, review of, 427.
Hulke, J. W., on iridectomy in glaucoma, 1069.
Humerus, fracture of anatomical neck of, 118.
Humphrey, G. M., M.D., on coagulation of blood in venous system during life, 511.
Hunter, John, memorial to, 765.
Hutchinson, Dr., on a friction sound of the peritoneum, 93.
on syphilitic infantile iritis, 1075.
Hydatid tumor of right ventricle of heart, 754.
Hydatid and abscess of liver, 756.
Hydatidiform or vesicular moles, 141.
Hydrochloric acid for dyspepsia, 1097.
Hydrocotyle Asiatica in chronic eczema, 1098.
Hydrostatic test in new-born children, 349.
Hypnotism, 380.
Hypertrophy of the prostate gland, 482.
Hypophosphites in phthisis, 545.
Hysteric paroxysm, chloroform in, 332.

ILEUM, intussusception of, 933.
Iliac artery, common, ligation of, 122, 874.
internal, aneurism of, 935.
Incontinence of urine, actual cautery in, 453.
Indian hemp, observations on, 739.
Infants, suction mode of rearing, 142.
Inman, T., M.D., on morning sickness, 718.
Insane, private asylums for, 376.
hospital appointments, 1133.
progressive paralysis of, 891.
Insanity in the United States, statistics of, 656.
Intestinal concretions formed of oat-hairs, 1115.
Intestines, incised wound of, 306.
Intoxication, as causing a type of disease, 890.
Intussusception of ileum, 933.
Introductory lectures and addresses by Wood, notice of, 443.
Inversion, chronic, of uterus, 139.
Iodine, cutaneous eruptions following use of, 743.
injections in ovarian cysts, 942.
in spina bifida, 1053.
Iodized oil of juniper-berries, 739.
Iridectomy in glaucoma, 1069.
Iris, exciting influence of light, cold, and heat on, 148.
Iritis of syphilitic infants, 1075.
Iron, stearate of, for chancre, 1097.
chloride of, in cutaneous diseases, 1101.
Irvin, W., M.D., on reduction of inverted uterus, 93.
Isaacs, C. E., M.D., on milky urine, 322.
necrological notice of, 957.
Itch, glycerin ointment in, 160.

JACKSON, DR., on discharge of the appendix cæci, 135.
on intussusception of ileum, 933.
Jacobi, A., M.D., on laryngismus stridulus, 333.
Japanese physicians, 760.
Jeaffreson, Dr., on suppression of urine, 130.
Jefferson Medical College, 381.
clinics of, 73, 1053.

Johnson, G., M.D., on Bright's kidney, 130.
Joints, treatise on diseases of by Bryant, review of, 810.
Joly, Dr., on coffee in strangulated hernia, 541.
Jordan, R. J., M.D., treatise on skin diseases by, notice of, 1025.
Just sentiments, 570.

KALEMA, properties of, 911.
Keating, Dr., on softening of the fornix and septum lucidum, 497.
Keith, Dr., on gelsemin, 743.
Keller, Dr., on tubercle of the lung, liver, and psoas abscess, 115.
on softening of brain, 306.
Kentucky Asylum for feeeble-minded children, 762.
Kerr, J. G., M.D., on spontaneous amputation of leg, 91.
Keyt, A. T., M.D., on mouth to mouth method in asphyxia, 332.
Kidney, abscess of, 757.
abnormal position of, 935.
albuminuria in one, saccharine urine in the other, 129.
Bright's, 116, 130.
movable, with spinal disease, 129.
tumor of, 758.
Kidneys, Bright's disease of, by Frerichs, review of, 962.
fused, 488.
Kletzinsky, Dr., on uroglaucine, 1108.
Kluge, J. P., M.D., on universal tuberculosis, 872.
Knee-joint, excision of, 1072.
Knees, lateral dislocation of, 310.
Knode, O. B., M.D., on bronchocele, 88.
on removal of patella, 486.
Kousso in tape-worm, 285.
Krackowizer, Dr., on excision of knee-joint, 1072.
Kurzck, Prof., on tannin as an antidote to strychnia, 744.

LABIUM, cellulo-fibrous tumor of, 266.
Labor, abnormal, version in, 1085.
Lagruan, Dr., on syphilitic tumors of tongue, 362.
Lancereaux, Dr., on abscess of the heart, 755.
Landerer, Dr., on the cornelian cherry, 534.
Lange, Dr., on quinia in acute rheumatism, 744.
Langer, J., M.D., on external cephalic version of the fœtus, 559.
Langier, Dr., on digital compression in aneurism, 1070.
Laryngeal affections, tracheotomy in, 171.
Laryngismus stridulus, observatious on, 333.
Laryngoscope, 759.
Larynx, fistulous ulcer of, 879.
scalds of, 501.
ventricles of, morbid growths of, 113.
Lawrence, Z., M.D., on morphia in acute sclerotitis and iritis, 358.
on sudden death, 877.
Lawrie, J. A., M.D., necrological notice of, 383.
Lebert, Prof., on embolie of cerebral artery, 1111.
Lectures, clinical, on the diseases of infancy and childhood by Best, notice of, 445.
on certain acute diseases, by Todd, review of, 801.
on diseases of women and children, by Bedford, notice of, 448.
on principles and practice of medicine, by Bennett, notice of, 449.
Lee, C. A., M.D., on chloroform in puerperal convulsions, 895.
Leg, weeping, 141.
Legouest, Dr., on coxo-femoral disarticulation, 939.
Leidy, J., M.D., on vesicating principle of Lytta vittata, 548.
Lenhossék, Prof., on pathology of spinal cords, 927.
Lens, crystalline, dislocation of, 1074.
Lente, F. D., M.D., of fracture of femurs by spasm, 1072.
Leriche, Dr., on borax in diphtheria, 345.
Leucorrhœa, arsenic in, 138.
Leudet, Dr., on hemorrhagic diathesis, 1082.
Levis, R. J., M.D., on a needle for wire suture, 176.
Ligation of femoral artery, 79, 703.
of popliteal artery, 79.
of primitive iliac, 122, 874.
Lime, saccharated, 547.
Lithotomy considered as a cause of death, 700.
lateral operation of, 84.
recto-vesical, 121.
Lister, J., M.D., on coagulation of the blood, 334.
Liver, fatty degeneration and acute softening of, 321.
hydatid tumor and abscess of, 756.
tubercle of, 115.
lungs and spleen, cancer of, 297.
sugar, researches on, 730.
Lizars, J., M.D., necrological notices of, 767, 958.

Lois, Dr., on fallacy of sources of arsenic in dead bodies, 350.
Lopez, A., M.D., on kousso in tape-worm, 285.
on milky urine, 286.
on opium as an antidote to belladonna, 285.
on rupture of spleen, 286.
on transverse fracture of patella, 286.
Lung, human, anatomy of, treatise on by Waters, notice of, 837.
cancer of, 115.
miliary tubercle of, 115.
Luschka, Prof., on the coccygeal gland of man, 734.
Lytta vittata, vesicating principle of, 548.

MacDonnell, R. L., M.D., on closure of the womb, 327.
Macilwain, G., M.D., memoir on strangulated hernia by, notice of, 450.
Maclachlan, C. F., M.D., on œsophageal fistule, 517.
Macleod, G. H. B., M.D., notes on surgery of Crimean war by, review of, 30.
Madder, coloration of fœtal bones by, 1093.
Maine Medical School, 186.
Maisonneuve, Dr., on naso-pharyngeal fibrous polyps, 122.
Malgaigne, J. F., M.D., treatise on surgical anatomy by, notice of, 822.
Malpractice and medical evidence, treatise on by Elwell, notice of, 832.
Man, moral and physical, 565.
Mania, homicidal, 133.
Mantigozza, Dr., on the properties of erythroxylon coca, 341.
Marcé, L. V., M.D., on hypochondriacal delirium, 716.
on water in the brain, 1107.
Marcy, L. M., M.D., on fatty degeneration and softening of liver, 321.
Martin, S., M.D., on ranunculus ficaria, 154.
Martini, Dr., on santonine in nervous affections of the eye, 737.
Marriages of consanguinity, 567.
Mascan, Dr., on action of salts on circulating red corpuscles, 143.
McGrie, Dr., on estimation of sugar in diabetic urine, 918.
McIlvaine, R. R., M.D., on cod-liver oil, 1102.
McKinley, Dr., on a case of rape, 165.
Medical classes in Canada, 381.
journals recently established and suspended, 382, 565, 764, 955, 1130.
Medical institutions in Philadelphia, 193.
jurisprudence, elements of, by Dr. Beck, review of, 433.
literature of Philadelphia, 385.
schools, changes in, 564, 955, 1131.
society of Pennsylvania, proceedings of, 763.
students in Philadelphia, 186.
students and graduates in United States for 1859–60, 564.
Medicine, ancient, 1125.
institutes of, treatise on by Payne, notice of, 841.
Medicines, action of in system, treatise on by Headland, notice of, 71.
Medullary fungus of pineal glands, 751.
Meigs, J. A., M.D., appointment of, 955.
on fragmentary skull, notice of, 73.
Meigs, J. F., M.D., on chronic gastritis, duodenitis and colitis, 95.
on transposition of arteries, 1113.
Melanosis, universal, 488.
Meli, Dr., on piperin in intermittent fever, 1098.
Memorial to John Hunter, 765.
Menorrhagia, arsenic in, 138.
Menses, retained, from imperforate os uteri, 897.
Mental diseases, diagnosis and prognosis of, 1077.
Mercury, iodide of the chloride of, in skin diseases, 535.
injurious effects of, treatise on by Habershon, review of, 632.
Mesentery and duodenum, cancer of, 293.
Messer, J. C., M.D., on the prostate gland in old age, 940.
Metcalfe, J. C., M.D., on occlusion of the os uteri during labor, 719.
Metcalfe, S. L., M.D., treatise on caloric by, notice of, 1019.
Michana, Dr., on tibio-tarsal amputation, 505.
Michel, M., M.D., on pathology of the pituitary body, 750.
Microscope, notes on how to work with, by Beale, notice of, 1024.
Microscopical diagnosis, treatise on by Van Düben, notice of, 263.
Middeldorpf, Prof., on gastric fistule, 708.
on œsophageal polyps, 503.
Midwifery practice, records of, 137.
statistics of, 893.
Milky urine, 286, 322.
Missionary Society's hospital at Canton, 1129.
Mitchell, S. W., M.D., on cancer of the liver, lungs, and spleen 297.

Moir, J., M.D., on retroflexion of uterus, 1088.
Mole, versicular, 141.
Möller, Dr., on formation of suggillations, 1109.
Monnerat, Dr., on a powder for chronic coryza, 347.
Montgomery, W. F. H., M.D., necrological notice of, 383.
Morphia, antiphlogistic properties of, 358.
 in puerperal convulsions, 723.
Morton, W. T. G., M.D., resolutions for testimonial to, 569.
Mosel-Lavallie, Dr., on dislocation of the clavicle, 314.
Mott's surgical clinics, report of by Francis, notice of, 839.
Mouth to mouth method in asphyxia monatorum, 332.
Muller, Prof., on composition of cerebral substance, 747.
 on smooth muscular fibres in orbit of man, 533.
Muscles, bronchial, paralysis of, 884.
Muscular fibres, smooth, in orbit of man, 533.
 motions, as affected by cerebellar hemorrhage, 319.
Mütter, Prof., age of, 380.
 discourse on life of, by Pancoast, notice of, 189.
Myeloid tumors, 877.

NASO-PHARYNGEAL POLYPS, new operation for, 122.
 removal of, 702.
Nature in disease, treatise on by Bigelow, notice of, 643.
Naval medical appointments, 763.
Neck, serous cyst of, 81.
Needle for the wire suture, 176.
Neff, C., M.D., appointment of, 955.
Neuralgia, cyanide of potassium in, 535.
 of the stump, 283.
Newbigging, P., M.D., on epilepsy in the case of Prof. Alison, 316.
Newman, W., M.D., on management of placenta, 893.
New Orleans Medical News and Hospital Gazette, 571.
New York College of Veterinary Surgeons, 187.
Nichols, W. C., M.D., on digital compression in aneurism, 1070.
Nightingale, Florence, 374.
 notes on nursing by, notice of, 1015.
Nitro-benzine, toxicological remarks on, 548.
Nunnely, F., M.D., treatise on organs of vision by, notice of, 835.
Nuttall, T , M.D., necrological notice of, 191.

OBSTETRIC CATECHISM by Warrington, notice of, 70.
Œsophageal fistule, 517.
Œsophagus, polyps of, 503.
Ogier, T. L., M.D., on ligation of femoral artery, 703.
Ogle, J., M.D., on ophthalmoscope in diagnosis of nervous affections, 888.
 on softening and ecchymosis of pons varolii and medulla oblongata, 931.
Oils, ozonized, 156.
Oleum morrhuæ, rajæ, and squali, researches on, 150.
Ollier, L., M.D., on reproduction of bone, 904.
Ophthalmia of new-born infants, 903.
 collyrium for, 345, 724.
 purulent, of infants and children, 879.
Ophthalmoscope in diagnosis of nervous affections, 888.
Opium as an antidote to belladonna, 285.
 caffein as an antidote to, 918.
Oregen, Dr., on rupture of right auricle of heart, 132.
O'Reilly, J., M.D., on anatomy and physiology of placenta, notice of, 1024.
O'Rorke, Dr., on oil of aleurites tribola, 348.
Orr, R. S., M.D., on ignorance of pregnancy up to time of delivery, 894.
 on a weeping leg, 141.
Orton, J. C., M.D., on cancer of stomach, 1083.
Osteitis, morbid actions constituting, 936.
Os uteri, occlusion of, during labor, 719.
Otto, Prof., on action of medicines on the mental faculties, 1106.
Ovarian tumor simulating pregnancy, 331.
 tumors, account of remarkable, 478.
  diagnosis of, 330.
  iodine injections in, 942.
Ovariotomy, report on, 502.
 origin of, 1028.
 statistics of in Ohio, 127.
Owen, R. D., footfalls on the boundary of another world by, review of, 615.
Ozanam, Dr., on bromine in pseudo-membranous affections, 161.
 on formula for solution of bromine, 744.

Packard, J. H , M.D., on cancer of bladder, 493.
on perforation of aortic valves, 932.
on pyogenic or suppurative diathesis, 99.
on stricture of rectum, 934.
Paget, J., M.D., lectures on surgical pathology by, notice of, 70.
Pancoast, Prof., discourse on life of Mütter by, notice of, 189.
Pancreas, a little-known function of, 528.
Paralysis, diagnosis and treatment of special forms of, 708.
intermittent phenomena resembling, 889.
of the bronchial muscles, 884
of the inferior rectus of eye, 477.
progressive, of the insane, 891.
Parisian doings, 570.
Parrish, E., M.D., treatise on practical pharmacy by, notice of, 69.
Parturition, impeded, obstetric binder and position in, 521.
Patella, removal of, 486.
transverse fracture of, 286.
Pathological Society's Proceedings, 93, 291, 488.
Pathology, cellular, Virchow's views on, 518.
of the cerebellum, 513.
surgical, lectures on by Paget, notice of, 70.
Paul, Dr., on lead poisoning on the product of conception, 1091.
Payne, M., M.D., treatise on institutes of medicine by, notice of, 841.
Peacock, T. B., M.D., on contraction and obliteration of aorta, 713, 753.
on phthisis of French mill-stone makers, 514.
on rapidly fatal anæmia, 134.
Peake, H., M.D., on coup de soleil, 859.
Peaslee, E. R., M.D., on displacements of the uterus, 722.
Pelikan, Dr., on tanghinia venenifera, 165.
Pelithan, Dr., on general phlegmon of abdomen, 757.
Pelvi-peritonitis, treatment of, 140.
Penghawar Djambi, observations on, 736.
Pennsylvania Hospital appointments, 763.
training school for feeble-minded children, 762.
Pepper, W., M.D., appointment of, 763.
Pepsin in vomiting of pregnancy, 739.
Peritoneum, friction sound of, 93.
Perspiration, excessive, of feet, 540.
Pescheux, Dr., on injection of sulphate of atropine in tetanus, 738.
Peugnet, Dr., on cancer of lung, 1115.
Pharmacopœia of United States, revision of, 381, 761.
Pharmacy, practical, treatise on by Parrish, notice of, 69.
Philadelphia anatomical rooms, 381.
hospital, appointments to, 955.
clinics of, 266, 453, 1133.
museum of, 1133.
lunatic asylum, 186.
medical institutions of, 193.
Philipot, H. J., M.D., on exfoliation of skull, 707.
Phillips, G. W., M.D., on gangrene of uterus and placenta, 72.
Phlegmon, general, of abdomen, 757.
Phlegmasia dolens, 1089.
Phosphates, syrup of, in stomatitis materna, 89.
Phosphorus, detection of, 549, 1110.
in paralysis of muscles of eye, 345.
Phthisis, influence of warm climates on, 887.
hypophosphites in, 545.
of French mill-stone makers, 514.
radical cure of, 955.
Physician's visiting list for 1860, notice of, 72.
Pineal gland, medullary fungus of, 751.
Pinel, a biographical study, notice of, 828.
Piperin in intermittent fever, 1098.
Pirrie, Dr., on puerperal convulsions, 895.
Pitha, Dr., on inflammation of sinuses of dura mater, 1073.
Pituitary body, pathology of, 750.
Placenta, management of, 893.
anatomy and physiology of, by Dr. O'Reilly, notice of, 1024.
delivery of, 1086.
Planchon, Dr., on globularia alypum, 534.
Pneumothorax, and aneurism of aorta, 1113.
Poisoning by arsenic, 747, 1111.
arsenical paper, 550.
cyanide of potassium, 351.
strychnia, 749.
Poisons, metallic, electrolysis as a means of detecting, 747.
Polyp, desmoid, 367.
of rectum, 84.
hemorrhage as a sign of, 333.
the cause of intussusception of ileum, 933.
Polyps, naso-pharyngeal, 122, 702.
of œsophagus, 503.
of uterus, 901.
Polypus-shaped desmoid, 367.
Popliteal artery, ligation of, 79.

Potassium, cyanide of, in neuralgia, 535.
iodide of, in cerebral affections of children, 527.
poisoning by, 351.
Practice, physicians' hand-book of, by Drs. Elmer and Elsberg, notice of, 71.
Pregnancy, can a woman remain ignorant of, 894.
and abdominal tumors, difficulty of diagnosis between, 523.
sickness of, 136.
Price, Dr., on excision of hip-joint, 706.
Prichard, Dr., on epilepsy, 930.
Prize offered to students of United States, 566.
of Paris Academy of Medicine, 566.
questions, 188.
Procidentia uteri, 1065.
Prostate gland, abscess of, 460.
condition of, in old age, 940.
hypertrophy of, 492.
Propylamin, remarks on, 544.
Prurigo, chelidonium majus in, 348.
Psoas abscess, 115.
Puerperal convulsions, chloroform in, 895.
digitaline in, 160.
injections of morphia in, 723.
treatment of, 895.
Pus, green or blue color of, 162.
Pyogenic or suppurative diathesis, 99.

QUAIN, R., M.D., on the hypophosphites in phthisis, 545.
Quinia, sulphate of. in acute articular rheumatism, 744.
in croup, 903.

RAMSBOTHAM, F., M.D., on chronic inversion of uterus, 139.
Ranking, W. H., M. D., on pneumothorax and aneurism of aorta, 1113.
Ranunculus ficaria, observations on, 154.
Rape. case of, committed on a child, 164.
Records of midwifery practice, 137.
Recto-vesical lithotomy, 121.
Rectum, polyp of, 84.
stricture of, 934.
Rectum and anus, treatise on by Ashton, notice of, 1021.
Regnoldi, G., M.D., necrological notice of, 191.
Reed, Dr., on Bright's kidney, 116.
Reeves, J. E., M.D., treatise on enteric fever by, review of, 57.
Renal abnormity, cases of, 757.
calculus, sequelæ of, 1067.
Retina, apoplectic affections of, 1081.
Retro-uterine hematocele, treatise on by Voisin, review of, 817.
Retroversion of uterus in pregnancy, 325.
Retzius, A., M.D., necrological notice of, 707.
Reynolds, J. B., M.D., on paralysis from diphtheria, 710.
Reyssie, Dr., on sambucus nigra in dropsy, 344.
Rhamnus alaternus as an antigalactic, 159.
Rheumatism, acute articular, quinia in, 744.
Riberi, Dr., on digital compression in aneurism, 311.
Richardson, Prof. T. G., M.D., on concussion of brain, 647, 649.
on compound fracture of skull, 651.
on dislocation of head of femur into thyroid foramen, 646.
on subnitrate of bismuth in burns and scalds, 655.
Ricord, Dr., on stearate of iron for chancres, 1097.
Rigden, Dr., records of midwifery practice by, 137.
Robin, C., M.D., on the capillaries of the brain, 149.
Roche, Dr., on cyanide of potassium in neuralgia, 535.
Rokitansky, Prof., on flexions of the uterus, 522.
Rolleston, I., M.D., on pathology of the cerebellum, 513.
Rome, ancient, medical studies on by Rouyer, review of, 790.
Roux, J., M.D., on hip-joint amputation, 880.
on secondary amputations after gunshot wounds, 704.
Rouyer, J., M.D., medical studies on ancient Rome by, review of, 790.
Roy, G. G., M.D., on procidentia uteri, 1065.
Rupture of the uterus during pregnancy, 721.

SACCHARATED LIME, 547.
Sambucus nigra in dropsy, 344.
Sands, Dr., on rupture of aortic valves, 131.
Sanguinaria canadensis, properties of, 542.
Sansom, Dr., on intestinal concretions, 1115.
Santonine in nervous affections of eye, 737.
Scalds of larynx, 501.
Scanzoni, Prof., on morphia in puerperal convulsions, 723.
Scapula, surgical neck of, fracture of, 882.

Schauenstein, Dr., on poisoning by cyanide of potassium, 351.
Scherer, Prof., on detection of phosphorus, 1110.
Schottin, Dr., on hydrochloric acid for dyspepsia, 1097.
Schiller, as a physician, 562.
Schmidt, Dr., on absence of bladder, 758.
on animal starch, 148.
Schneider, Dr., on poisoning by strychnia, 749.
Schroff, Dr., on hellebore, 151.
Schweinfurt green, dangers of, 1109.
Scirrhus of breast, 281.
Sclerotitis, acute, morphia in, 358.
Scrofulous diathesis, 885.
Scrotum, rare variety of tumor of, 125.
Scuh, Prof., on iodine injections in ovarian cysts, 942.
Scwartz, Dr., on Chinese medicines, 346.
Sédillot, Dr., on actual cautery in epithelial cancer, 126.
Selenium palustre in epilepsy, 348.
Senile gangrene, 472.
Serous cyst of neck, 81.
Serre, Dr., on digitaline in puerperal fever, 160.
Sheetz, L. D., M.D., on delivery of placenta, 1086.
Sickness, morning, as a symptom, 718.
of pregnancy, 136.
Sigmund, Prof., on stricture of urethra, 881.
Simon, G., M.D., on desmoid polyp, 367.
Simpson, J. G., M.D., on acupressure, 308, 552.
Sinapisms, glycerin in preparation of, 738.
Skin diseases, chloride of zinc in, 912.
treatise on by Jordan, notice of, 1025.
Skull, compound fracture of, 651.
deformed and fragmentary, description of by Meigs, notice of, 73.
exfoliation of, 707.
Small-pox in Scotland, 515.
Smith, A., M.D., records of midwifery practice by, 138.
Smith, H., M.D., treatise on hemorrhoids by, notice of, 644.
Smith, S., M.D., on ligation of primitive iliac, 874.
Softening of fornix and septum lucidum, 497.
Solanaceæ in tenesmus, 348.
Solly, Dr, on aneurism of aorta, 132.
Southam, G., M.D., on recto-vesical lithotomy, 121.
Southern medical students and schools, 378.
Spence, G. W,, M.D., on preparing and applying chloride of zinc, 542.
Spencer, P. C., M.D., necrological notice of, 573.
Spina bifida, iodine injections in, 1053.
Spinal cord, pathology of, 927.
Spine, fracture of, 492.
Spleen, rupture of, 286, 756.
Stabler, Dr., on propylamin, 544.
Staphyloma, ligature treatment of, 883.
Starch, animal, 148.
Stellway, Prof., on purulent ophthalmia of children, 879.
Stillé, A., M.D., treatise on materia medica and therapeutics by, review of, 588.
Still-born children, resuscitation of, 142.
Stomach, cancer of, 1083.
Stomatitis materna, 89.
Stone, W., M D., on ligation of primitive iliac, 122.
Stone, importance of early diagnosis of, 352.
in the bladder, 312, 466, 875.
Strangulation, studies on, 925.
Stricture of rectum, 934.
urethral, 467.
treatment of, 315, 881.
Strube, Dr., on uva ursi as a parturifacient, 136.
Strychnine, poisoning by, 749.
tannin, an antidote to, 744.
Students in Philadelphia, 565.
in London and Paris, 565.
Stump, neuralgia of, 283.
Styria, arsenic eaters of, 920.
Subungual exostosis, 876.
Sucquet, J. F., M.D., on circulation of blood, treatise on by, notice of, 840.
Sugar in the glucogenous matter of liver, 163.
in urine, tests for, 161.
mode of estimating quantity of, in diabetic urine, 918.
Suggillations, formation of, 1109.
Suicide in Massachusetts, 567.
Surgery, dental, system of by Tomes, review of, 414.
of Western Nations, treatise on by Hobson, notice of, 72.
principles and practice of by Druitt, notice of, 1022.
Surgical pathology, lectures on by Paget, notice of, 70.
Sydenham Society's publications, notice of, 645, 1133.
Sylvester, Dr., on resuscitation of still-born children, 142.
Sympathetic nerve, experiment on cervical portion of, 531.
Syncope from surgical hemorrhage, treatment of, 703.

Syphilis, constitutional, iodide of ammonium in, 349.
Syphilitic aphonia, 882.
iritis, 1075.
tumors of tongue, 362.
Swayne, J. G., M.D., on fibrous tumor of uterus, 901.

TADDEI, PROF., necrological notice of, 959.
Taft, J., Prof., treatise on operative dentistry by, notice of, 414.
Tanghinia venenifera, properties of, 165.
Tannin, an antidote to strychnia, 744.
glycerole of, 898.
in uterine affections, 539.
Tape-worm, kousso in, 285.
Tardieu, J., M.D., on granulations of uterus, 328.
on strangulation, 925.
Tavignot, Dr., on phosphorus in paralysis of muscles of eye, 345.
Taxis in strangulated hernia, 209.
Teale, Dr., on stone in the bladder, 312.
Tenesmus, solanaceæ in, 348.
Testicle, cystic disease of, 76.
Tetanus, curare in, 344.
Therapeutics and materia medica, treatise on by Stillé, review of, 588.
Thomas, T. G., M.D., on prevention of post-partum hemorrhage, 324.
on treatment of post-partum hemorrhage, 520.
Thompson, H., M.D., on importance of early diagnosis and treatment of vesical calculus, 352.
on internal incision in urethral stricture, 315.
Thompson, O., M.D., on ozonized oils, 156.
Thoracic duct, urea in fluids of, 533.
Thudichum, J. L. W., M.D., on liver-sugar, 730.
on gall-stones, 177.
Thuiæ tinctura, in bad smells of nose, 915.
Thymus gland, enlargement of, 1091.
Tibial artery, anterior, aneurism of, 79.
Tibio-tarsal amputation, 505.
Tic douloureax, operation for, 506.
Tilt, Dr., on pelvi-peritonitis, 140.
Todd, R. B., M.D., clinical lectures on acute disease by, review of, 801.
necrological notice of, 574.
Tomes, J., system of dental surgery by, notice of, 414.
Tongue, syphilitic tumors of, 362.
Toulmin, Dr., on phthisis, 713.
Toynbee, J., M.D., treatise on diseases of ear by, review of, 769.
Tracheotomy for foreign body, 1062.
Tracheotomy for laryngeal affections, 171.
in croup, 165, 505, 854.
Transactions of American Medical Association, notice of, 639.
of College of Physicians of Philadelphia, notice of, 1026.
of New Jersey State Medical Society, notice of, 1027.
of Pennsylvania State Medical Society, notice of, 446.
Trichina spiralis, fatal case of, 884.
Triplett, W. H., M.D., on renal calculus, 1067.
Tubercle, miliary of lung, 115.
of liver, 115.
Tuberculosis, universal, 872.
Tumor, cellulo-fibrous, of labium, 266.
colloid, of abdomen, 866.
hydatid, of heart, 754.
of liver, 756.
in fourth ventricle of brain, 318.
of breast, 119.
of scrotum, 125.
Tumors, fibrous, of uterus, 899.
myeloid, 877.
ovarian, 478.
diagnosis of, 330.
syphilitic, of tongue, 362.
Typhoid fever, with penetrating ulcer, 305.

ULCER, fistulous, of larynx, 879.
Ulna, excision of, 1073.
Urea, in chyle and lymph, 339.
in fluids of thoracic duct, 533.
Urethra, stricture of, 315, 467, 881.
Urine, diabetic, estimation of quantity of sugar in, 918.
incontinence of, actual cautery in, 453.
milky, 286, 322.
sugar in, modes of detecting, 161.
suppression of, 130.
Uroglaucine, 1108.
Uterine catheterism, to produce abortion, 325.
Uterus, affections of, tannin in, 539.
and placenta, gangrene of, 721.
closure of, treatment of, 327.
diseases of, 1116.
displacements of, 722.
fibrous tumors of, 899.
granulations of, 328.
inflexions of, 522.
inversion of, 93, 139.
polyps of, 901.
retroflexion of, 1088.
retroversion of, in pregnancy, 325.
rupture of, during pregnancy, 721, 1087.

Uva ursi as a parturifacient, 136.

VAGINITIS, glycerole of tannin in, 898.
Valerian, in infantile convulsions, 1093.
Van Düben's treatise on microscopical diagnosis, translated by Bauer, notice of, 263.
Variola, attempts to produce in cow, 716.
chlorine lotions in, 537.
Veiel, Dr., on chloride of zinc in skin diseases, 912.
Veins, varicose, button-suture in, 877.
Verneuil, Dr., on scrotal tumor, 125.
Vesico-vaginal fistule, new operations for, 313.
Veterinary science in Europe, 569.
Vinke, Dr., on penghawar djambi, 736.
Virchow, Prof., appointment of, 186.
on cellular pathology, 518.
on flexions of uterus, 523.
Vision, organs of, treatise on, by Nunneley, notice of, 835.
Vogt, W., M.D., on antipyretic remedies, 914.
Voisin, A., M.D., treatise on retro-uterine hematocele by, review of, 817.
Voss, A. L., M.D., on tracheotomy in croup, 504.

WAGNER, R., M.D., experiments on cervical portion of sympathetic by, 531.
Warrington, J., M.D., obstetric catechism by, notice of, 70.
Waters, A. T. H., M.D., treatise on anatomy of human lung by, notice of, 837.
Watson, J., M.D., on fistulous ulcer of larynx, 879.
Watson, J. M., M.D., on influence of mother's mind on fœtus in utero, 521.
Weeping leg, 141.
Wells, W. S., M.D., an epitome of Braithwaite's Retrospect by, notice of, 453, 1025.
West, C., M.D., lectures on diseases of infancy and childhood by, notice of, 445.
West, C., M.D., necrological notice of, 957.
on cerebral symptoms, without cerebral disease, 320.
on mental peculiarities and disorders of childhood, 526.
Wheelock, Dr., on acute rheumatism, 1082.
Wiedenhold, Dr., on tests for sugar in urine, 161.
Williams, Dr., on carbonate of ammonia in bronchitis, 539.
Williams, R. C., M.D., necrological notice of, 958.
Williamson, G., M.D., on excision of ulna, 1073.
Wilson, Dr., on instrument for inflating lungs of asphyxiated infants, 903.
Winslow, F., M.D., treatise on obscure diseases of brain by, review of, 1003.
Wood, A., M.D., on small-pox in Scotland, 515.
on tumor in fourth ventricle of brain, 318.
Wood, G. B., M.D., dinner to, 765.
introductory lectures and addresses by, notice of, 443.
portrait of, 567.
Wood, J. R., M.D., on supra-pubic operation of lithotomy, 875.
Wood, W., M.D., on progressive paralysis of insane, 891.
Woorara, observations on, 343.
Wörz, Dr., intermittent lameness from emboli, 558.
Wound of heart, 496.
Wrist, ganglions of, 82.
Wurtz, A., M.D., on urea in chyle and lymph, 339.
on urea in fluids of thoracic duct, 533.
Wythes, J. H., M.D., on remarkable ovarian tumors, 478.

YEAR-BOOK of American contributions to medical science and literature, 1130.

ZINC, chloride of, in skin diseases, 912.
mode of preparing and applying, 542.

